# Synthesis and Properties of Polyarylene Ether Nitrile and Polyphenylene Sulfone Copolymers

**DOI:** 10.3390/polym18020159

**Published:** 2026-01-07

**Authors:** Azamat Zhansitov, Kamila Shakhmurzova, Zhanna Kurdanova, Azamat Slonov, Ilya Borisov, Elena Rzhevskaya, Ismel Musov, Artur Baykaziev, Svetlana Khashirova

**Affiliations:** Center for Progressive Materials and Additive Technologies, Kabardino-Balkarian State University Named After H.M. Berbekov, 360004 Nalchik, Russiaazamatslonov@yandex.ru (A.S.); boril@ips.ac.ru (I.B.);

**Keywords:** polyphenylene sulfone, polyarylene ether nitrile, polycondensation, tensile strength, crystallization

## Abstract

Copolymers of polyphenylene sulfone and polyarylene ether nitrile were synthesized using nucleophilic polycondensation. 2,6-difluorobenzonitrile (DFBN), 4,4′-dihydroxybiphenyl, and 4,4′-dichlorodiphenyl sulfone were used as monomers. The structure of the obtained copolymers was confirmed by means of IR spectroscopy, and their solubility in various solvents was studied. Thermal properties were studied using differential scanning calorimetry (DSC) and thermogravimetric analysis, as well as a set of basic mechanical properties. It was found that both thermal stability and glass transition temperature are virtually independent of the copolymer composition, while samples with a DFBN monomer content of more than 75% exhibit a melting peak in the region of 357 °C on the DSC curves, indicating an increase in the degree of crystallinity, accompanied by a deterioration in the solubility of these polymers. With increasing DFBN content, a uniform increase in elastic modulus is observed, and both bending and tensile strength increase significantly. However, the introduction of DFBN segments into the polyphenylene sulfone structure leads to a decrease in impact strength.

## 1. Introduction

Membrane separation technologies occupy a leading position in modern separation processes due to a number of significant advantages over traditional methods: high energy efficiency, technological simplicity, environmental safety, and process scalability [1]. These technologies have been successfully integrated into the pharmaceutical industry [2], biotechnology [3], fuel cell systems [4], and, in particular, into water and wastewater treatment processes [5,6].

Polymer membrane materials used on an industrial scale are represented by a wide range of highly effective polymers, including polysulfone (PSF) [7,8,9,10,11,12], polyethersulfone (PES) [13,14], polyvinylidene fluoride [15,16,17,18], polyimides [19,20,21,22], and polyacrylonitrile [23,24]. The listed materials are characterized by an optimal combination of mechanical characteristics, physical and chemical resistance and thermal stability, which ensures their wide application [25,26].

Among polymer membranes, polyarylsulfones, including PSF, PES, and polyphenylene sulfone (PPSU), have found wide application as membranes for various applications in gas separation, micro- and ultrafiltration, hemodialysis, and other processes due to their high mechanical strength, chemical inertness, thermal stability, and hydrolytic resistance to superheated steam [12,27]. The results of studies on the synthesis and characterization of new copolymers based on polyarylsulfones, their chemical modification, and functionalization to obtain membranes with a given set of properties are regularly published [11,27,28,29,30].

PPSU is a rather promising polymer from the polyarylsulfone series for the production of various types of membranes, which can withstand treatment with superheated steam without losing its original properties for up to 2000 cycles [28]. It is known that PPSU is used as a membrane-forming material in various filtration processes such as nanofiltration of organic media [31,32], ultrafiltration of aqueous media [33,34,35], and gas separation [36,37].

Polyarylene ether nitrile (PAEN) is a semicrystalline engineering polymer with an outstanding combination of performance properties. Due to its excellent mechanical properties, high thermal and radiation resistance, and exceptional physical and chemical stability [38,39,40], this material has found wide application in the automotive, electronics, defense, and aerospace industries. Studies have been conducted using various bisphenols to synthesize PAEN via nucleophilic aromatic polycondensation [39,41,42,43,44,45,46].

In the last decade, research interest in PAEN has significantly expanded in the context of membrane water treatment technologies [47], opening up promising avenues for its practical application in environmental technologies.

However, the practical use of PAEN in membrane technologies is associated with significant technological limitations. Being a semicrystalline polymer with an ordered macromolecular structure [48], PAEN exhibits extremely limited solubility in common organic solvents. PAEN dissolution is achieved exclusively in aggressive environments—concentrated mineral acids (sulfuric, trifluoroacetic) and toxic phenolic solvents [40,49], the use of which in technological processes for the formation of membranes via the phase inversion method is practically unacceptable from the point of view of industrial safety, environmental requirements and economic feasibility.

In view of the above limitations, chemical modification of PAEN by introducing polar functional groups into the polymer chain appears to be a promising approach. Thus, in [50], PAENS copolymers based on bisphenol A and 4,4′-dichlorodiphenylsulfone (DCDPS) together with 2,6-dichlorobenzonitrile (DCBN) were synthesized and their optical, thermal, mechanical, and dielectric properties were studied. It has been shown that with an increase in the proportion of DCDPS, the thermal properties improve, but the mechanical and dielectric properties are worse compared to PAEN. In [41], PAEN copolymers were synthesized and studied using the nucleophilic substitution method of DCBN with different molar ratios based on 4,4′-dihydroxybiphenyl (DHBP) and hydroquinone. Moreover, the obtained copolymers were dissolved in N,N-dimethylacetamide, N-methyl-2-pyrrolidone and dimethylformamide only upon heating. It was found that the glass transition temperature with an increase in the content of DHBP units increases to 216 °C and the melting temperature to 352 °C, and the tensile strength of the copolymers also increases. Similar results were obtained in [44,51], in which the thermal, mechanical and dielectric properties of PAEN-based films based on DHBP and DCBN were studied.

At the same time, given the results obtained in recent years on the fabrication and study of the properties of PPSU-based membranes [27,52,53], demonstrating the promise of their use, including in water purification, the synthesis of copolymers based on DHBP and DCDPS using 2,6-difluorobenzonitrile (DFBN) as a comonomer is of scientific interest.

The choice of this approach is determined by several factors. Firstly, copolymers of this structure have been little studied in the scientific literature and represent a promising class of materials combining the advantages of both PAEN and PPSU. Secondly, the use of materials of this structure for the fabrication of selective membranes has not been described in the scientific literature, opening up opportunities for producing new membranes with a unique combination of properties. Thirdly, the introduction of a polysulfone block into the PAEN structure solves the problem of polymer solubility in technologically acceptable aprotic dipolar solvents (N,N-dimethylacetamide, N-methyl-2-pyrrolidone, dimethyl sulfoxide) by reducing the degree of crystallinity. Moreover, the presence of nitrile groups is expected to increase the hydrophilicity and water absorption of the resulting membranes, while the use of DHBP will improve the mechanical and thermal properties compared to bisphenol A-based PAEN copolymers.

Thus, the aim of this study is to synthesize and characterize the basic physicochemical properties of PPSU copolymers with PAEN to determine the feasibility of their further use as materials for the fabrication of new polymer membranes for various applications.

## 2. Materials and Methods

### 2.1. Materials

2,6-difluorobenzonitrile (98%) was obtained from Yangzhou Tianchen Chemicals (Guazhou, China). 4,4′-dihydroxybiphenyl (99%) was obtained from Zhengzhou Alfa Chemical Co., Ltd. (Qinyang City, China). 4,4′-dichlorodiphenyl sulfone (≥99.5%) was purchased from Hebei Jianxin Chemical Co., Ltd. (Cangzhou, China). N,N-dimethylacetamide (≥99%), N-Methyl-2-pyrrolidone (≥99%), chloroform (≥99%), and methylene chloride (˃99%) were purchased from EKOS-1 (Moscow, Russia). Potassium carbonate (99+%) and styrene (98+%) were purchased from Reachem (Moscow, Russia). Tetrahydrofuran (99.9%) was obtained from RCI Labscan Limited (Bangkok, Thailand), dimethylformamide (≥99%) was obtained from Vecton (Saint Petersburg, Russia), and dichloroacetic acid (99+%) was obtained from Alfa Aesar (Haverrill, MA, USA). All of the materials were used as received without further purification.

### 2.2. Synthesis of PPSU and PAEN Random Copolymers

PPSU and PAEN random copolymers were synthesized by the nucleophilic aromatic substitution polycondensation in a 500 mL three-neck flask equipped with a nitrogen inlet, a mechanical stirrer, a Dean–Stark trap, and a reflux condenser. The method for obtaining PPSU is the same as in the article [54].

**Scheme 1:**
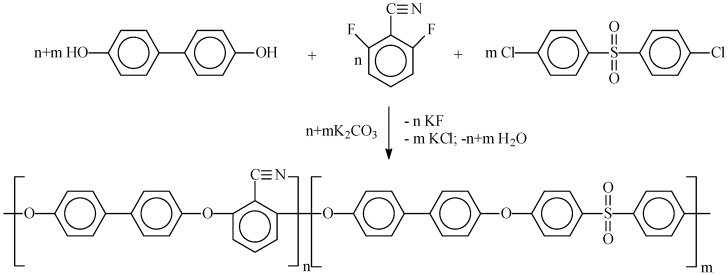
Synthesis of copolymer polyphenylene sulfone (PPSU) with polyarylene ether nitrile (PAEN).

Copolymer of PPSU with 2,6-difluorobenzonitrile was synthesized according to Scheme 1.

A typical preparation of PAEN copolymers is described below. DHBP (29.79 g, 0.16 mol), DFBN (5.56 g, 0.04 mol), DCDPS (36.01 g, 0.1256 mol), and K_2_CO_3_ (33.1 g, 0.24 mol) were added to a 500-mL three-necked flask. Then, N,N-dimethyl acetamide (450 mL) as reaction solvent was added. The reaction mixture was gradually heated to 165 °C for 4 h to distill the dimethylacetamide–water mixture. After the temperature reached 165 °C, the reaction mixture was allowed to proceed at this temperature for 6 h. After synthesis, the mixture was discharged, and the formed salts were filtered. The reaction solution was slowly poured into the water, acidified by oxalic acid. The precipitated polymer was filtered and washed 10 times with hot distilled water and dried in a vacuum oven at 160 °C for about 12 h. The ratios of monomers in the synthesis of polymers are given in Table 1.

### 2.3. Characterization and Methods

IR spectra were recorded on a Fourier spectrometer (Spectrum Two; PerkinElmer, Inc., Waltham, MA, USA) in the range of 4000–450 cm^−1^ with a spectral resolution of 0.4 cm^−1^. The glass transition temperature of the synthesized polymers was determined by differential scanning calorimetry (DSC) on a DSC 4000 instrument (PerkinElmer, Inc., Waltham, MA, USA). The quantity of 5–10 mg of the sample was sealed in an aluminum hermetic pan and heated to 400 °C at 10 °C/min under a flow of nitrogen (20 mL/min).

Thermogravimetric analysis (TGA) of the synthesized polymers was carried out on a TGA 4000 instrument (PerkinElmer, Inc., Waltham, MA, USA) under a flow of air (20 mL/min). at a heating rate of 10 °C/min in the range from 30 to 850 °C.

The melt flow index (MFI) of polymers was determined at a temperature of 380 °C and a load of 5 kg on a capillary viscometer (PTR-LAB-02, LOIP, Saint Petersburg, Russia).

Mechanical tests were carried out on a universal testing machine, CT-TCS 2000 (Gotech Testing Machine Inc., Taichung, Taiwan), at 23 °C. Impact tests were performed with and without a notch, by the Izod method on the instrument, GT-7045-MD (Gotech Testing Machine Inc., Taichung, Taiwan).

The solubility of the synthesized samples in various solvents was studied at 23 °C for 24 h for 5% polymer solutions.

The crystallinity of the synthesized powders was assessed by X-ray phase analysis using a D2 Phaser diffractometer (Bruker AXS, Karlsruhe, Germany) with monochromatic CuKα radiation (λ = 1.5406 Ǻ) in the range of 10–90°.

## 3. Results and Discussion

### 3.1. Study of the Structure of Copolymers by IR Spectroscopy

**Figure 1:**
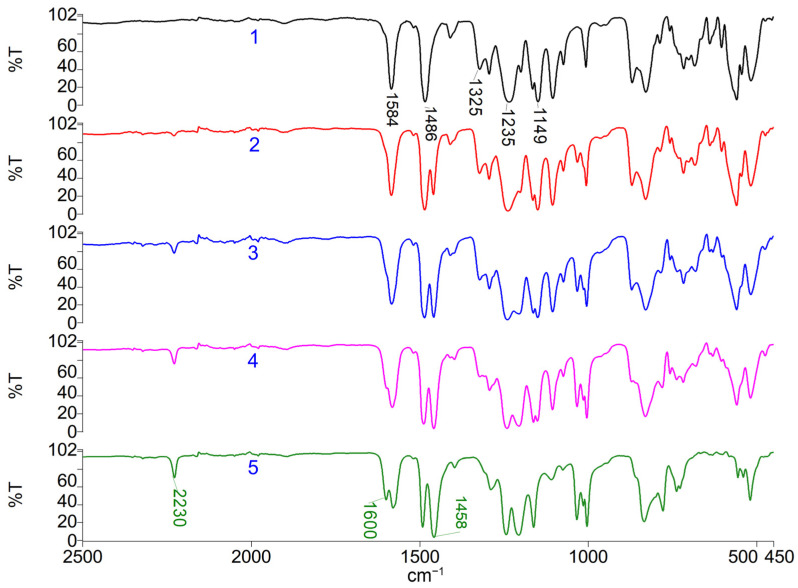
IR spectra: 1—PPSU; 2—PPSU/PAEN 75/25; 3—PPSU/PAEN 50/50; 4—PPSU/PAEN 25/75; 5—PAEN.

The structure of the synthesized copolymers was studied using IR spectroscopy. The absorption bands in the spectra of the synthesized polyphenylene sulfone (PPSU) correspond to those previously described for this polymer [30]. The skeletal vibrations of the C–C aromatic bonds appear as bands with maxima at 1584 and 1486 cm^−1^ (Figure 1). In the studied copolymers, a bifurcation of these bands is observed, with additional peaks appearing in the region of 1600 and 1458 cm^−1^, the intensity of which increases consistently with increasing DFBN comonomer content. PPSU also exhibits highly characteristic absorption bands in the 1325 cm^−1^ and 1149 cm^−1^, due to symmetric and antisymmetric vibrations of the SO_2_ group, respectively. Moreover, the intensity of these bands decreases naturally with a decrease in the proportion of 4,4′-dichlorodiphenylsulfone in the copolymers. The intense band at 1235 cm^−1^ is associated with asymmetric stretching vibrations of the C–O group [55].

The absorption band at 2230 cm^−1^ is characteristic of the symmetrical stretching vibration of the CN group [46]. The intensity of this band can also be used to estimate the percentage of DFBN in the copolymers.

### 3.2. Thermal and Rheological Properties

In order to obtain copolymers with specified molecular weight values, allowing their processing by injection molding and extrusion methods, and to minimize the effect of molecular weight values on the studied properties of the copolymers, the synthesis was carried out using the monomer equivalence rule according to the monomer ratios selected previously [54] for the synthesis of PPSU. As can be seen from the obtained MFI values (Table 1), these monomer ratios made it possible to obtain copolymers with similar molecular weight values, allowing the production of samples for testing mechanical properties by injection molding. In the majority of published studies on the synthesis of various PAEN copolymers, mechanical property tests were carried out on films obtained from solution [41,44,45,46,50,51].

**Figure 2:**
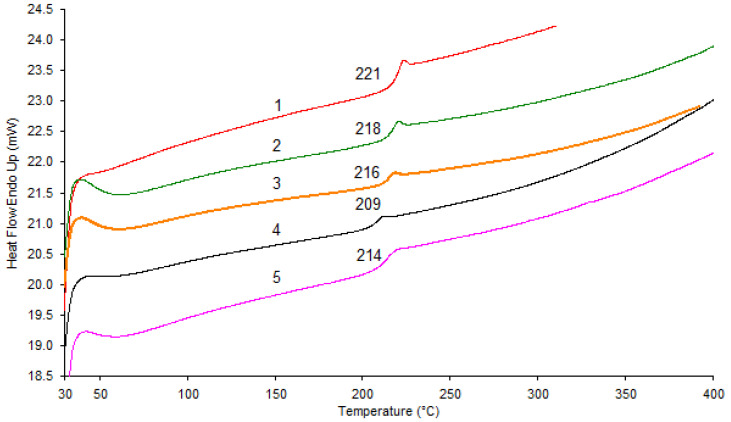
Differential scanning calorimetry second heating curves: 1—PPSU; 2—PPSU/PAEN 75/25; 3—PPSU/PAEN 50/50; 4—PPSU/PAEN 25/75; 5—PAEN.

As can be seen from the results presented in Table 2 and Figure 2, the glass transition temperatures determined during the second heating of the samples have similar values, with a tendency to decrease slightly with an increasing proportion of DFBN comonomer, which is consistent with previously obtained results in [41,44,50,51]. The lower glass transition temperature (209 °C) of the PPSU/PAEN 25/75 sample is associated with a lower molecular weight compared to the other samples, which is confirmed by higher melt flow index (MFI) values. The obtained values of the glass transition temperature of the PPSU homopolymer completely coincide with the passport values of the industrially produced PPSU Radel R-5000 brand [56].

**Figure 3:**
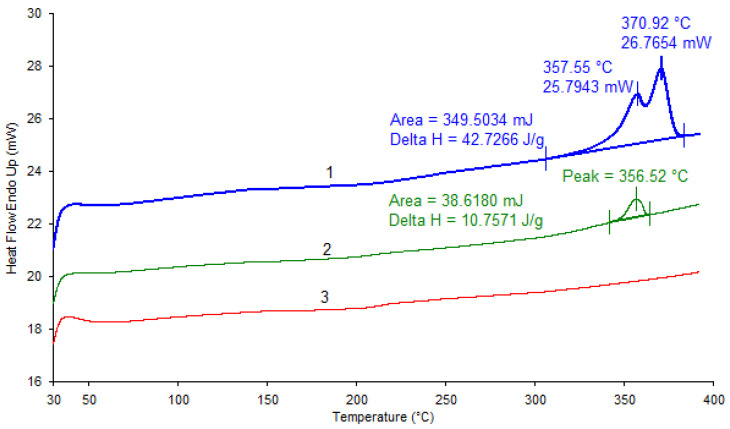
Differential scanning calorimetry first heating curves: 1—PAEN; 2—PPSU/PAEN 25/75; 3—PPSU/PAEN 50/50.

In the DSC curves of the first heating (Figure 3), only pure PAEN and the copolymer with 75% DFBN comonomer showed melting peaks in the DSC curves, indicating an increase in the degree of crystallinity of these samples. Moreover, the PAEN homopolymer has a fairly high melting point, manifested in the form of 2 melting peaks in the region of 358 and 371 °C. It is also worth noting that when cooling the samples, crystallization peaks were not observed, and upon reheating, only a phase transition corresponding to the glass transition was present on the DSC curves, which indicates a decrease in the degree of crystallinity of the samples after heat treatment above the melting point.

**Figure 4:**
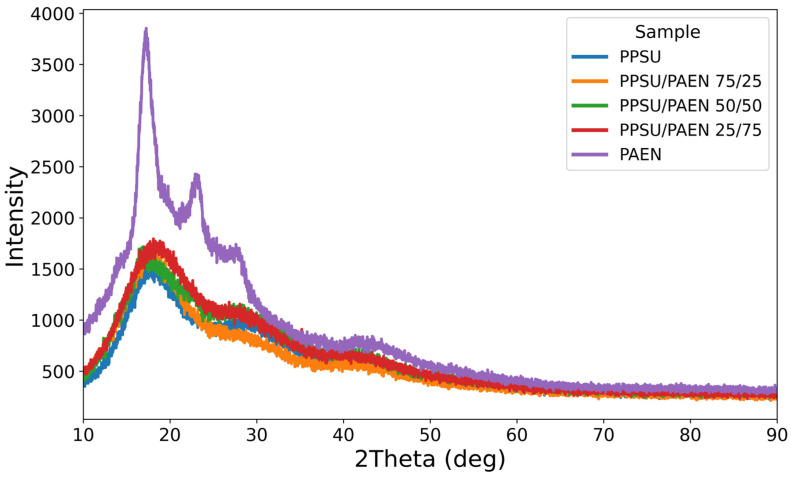
Wide-angle X-ray diffraction patterns of copolymers.

The results obtained by wide-angle XRD are consistent with the DSC analysis results. As can be seen in Figure 4, the curves of both PPSU and copolymer samples with different DCDPS contents have an amorphous halo, while the PAEN homopolymer exhibits narrow, intense long-range diffraction peaks at approximately 2θ ≈ 17° and 23° and a small peak at 2θ ≈ 29°, indicating the presence of a crystalline phase in this polymer, which is consistent with the results previously described for this polymer [44].

**Figure 5:**
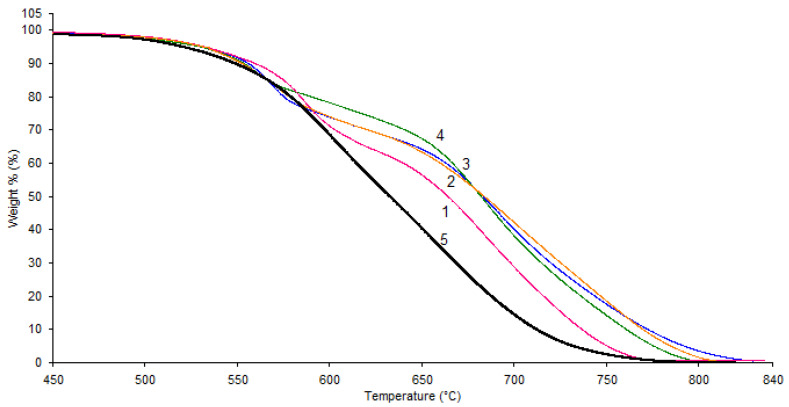
Thermogravimetric analysis curves: 1—PPSU; 2—PPSU/PAEN 75/25; 3—PPSU/PAEN 50/50; 4—PPSU/PAEN 25/75; 5—PAEN.

It is worth noting that the thermal stability of all samples was virtually identical (Figure 5), with the exception of the PAEN sample, which exhibited a slight decrease in thermal stability. Moreover, the TGA curves of the PPSU/PAEN 25/75 and PAEN samples, unlike the other samples, did not show a pronounced second stage of degradation.

### 3.3. Study of Solubility in Various Solvents

One of the main requirements for polymeric materials used in the manufacture of membranes (gas separation, filtration, etc.) produced from solution is their ability to dissolve in organic solvents. Good solubility is also important for polymeric materials used as coatings, including carbon and glass fibers, which improve adhesion between the fiber and the polymer matrix. The insolubility of PAEN homopolymers limits their application.

The solubility of the synthesized homopolymers and copolymers was studied for 24 h at room temperature, using both powder samples obtained during synthesis and pellets obtained from the melt after MFI analysis of these samples. The polymer solution concentration was 5%. As can be seen from the results presented in Table 3, the PAEN homopolymer samples were insoluble in all solvents used, and only the pellets swelled in dimethylacetamide and dimethylformamide. Reducing the DFBN content to 75% in the copolymer results in granulated samples becoming soluble after 24 h in solvents such as N-methyl-2-pyrrolidone and dimethylacetamide. Samples with 25 and 50% DFBN contents dissolve significantly better, as does the PPSU homopolymer. The improved solubility of granules obtained by melting, compared to the initial powder samples, may be due to the increased crystallinity of the powders obtained during synthesis, which prevents their dissolution, and the significant amorphization of the molten samples obtained after MFI analysis. This is confirmed by the results of DSC analysis, where no melting peaks are observed in the second heating cycle, and no peaks corresponding to the crystallization process are observed upon cooling the sample.

### 3.4. Mechanical Properties

Table 4 shows that increasing DFBN content leads to a uniform increase in elastic modulus, increasing by 25% for the PAEN sample. Both flexural and tensile strength also increase significantly. However, the introduction of DFBN segments into the PPSU structure leads to a decrease in impact strength, with PAEN exhibiting approximately half the impact strength of the original PPSU. Despite this, impact strength values, both notched and unnotched, remain quite high for all copolymer samples.

**Figure 6:**
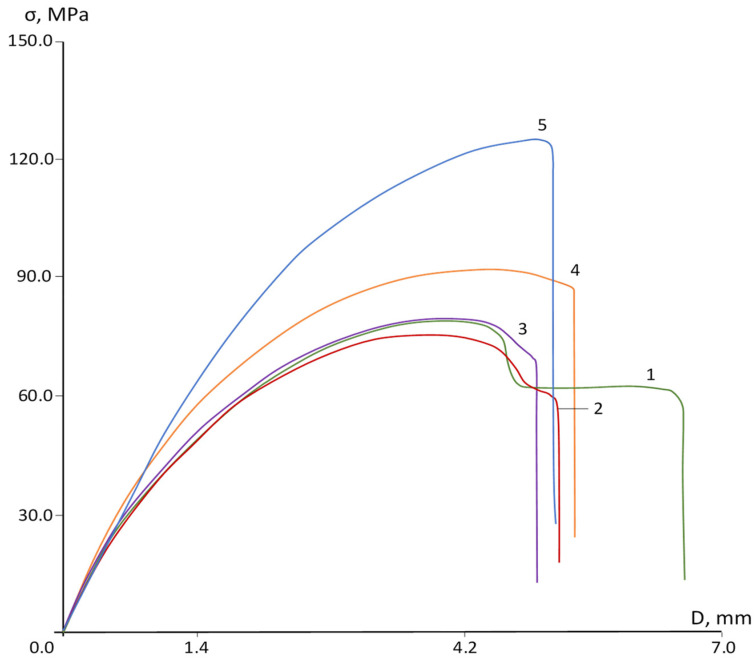
Deformation curves: 1—PPSU; 2—PPSU/PAEN 75/25; 3—PPSU/PAEN 50/50; 4—PPSU/PAEN 25/75; 5—PAEN.

The decrease in ductility is also confirmed by changes in the stress-strain curves of the studied materials. Figure 6 shows that the original PPSU exhibits a pronounced yield point and subsequent plastic deformation. The copolymer containing 25% DFBN as a comonomer also exhibits necking, but the magnitude of plastic deformation is significantly reduced. At 50% DFBN content, the yield strength is very weak, and the sample fails without forming a complete neck. A further increase in DFBN content to 75% in the copolymer is accompanied by an even greater decrease in ductility, and the PAEN itself exhibits a rather brittle fracture pattern.

The observed changes in properties may be due to the influence of the DFBN comonomer on the rigidity of the macromolecular chain, the nature of intermolecular interactions, and the molecular weight characteristics of the synthesized materials.

## 4. Conclusions

Thus, previously unstudied copolymers of PPSU and PAEN were synthesized using nucleophilic polycondensation.

It was found that the synthesized copolymers retain the high thermal stability and glass transition temperature characteristic of the PPSU homopolymer. Increasing the DPBN content in the copolymers to more than 75% leads to an increase in the degree of crystallinity of the polymer powders formed during synthesis, while after heating above the melting point, upon repeated heating, these copolymers do not exhibit melting or crystallization peaks in the DSC curves. At the same time, increasing the DPBN content leads to a uniform increase in the elastic modulus, with a significant increase in both flexural and tensile strength. However, the introduction of DPBN segments into the polyphenylene sulfone structure leads to a decrease in impact strength.

As expected, the introduction of a polysulfone block into the structure solves the solubility problem of the PAEN polymer based on DFBN and DHBP in organic aprotic dipolar solvents by reducing the degree of crystallinity. Thus, copolymers containing 25 and 50% DFBN, like the PPSU homopolymer, dissolve in N,N-dimethylacetamide, N-methyl-2-pyrrolidone, and dimethylformamide at room temperature, which significantly expands their potential applications, including the production of separation membranes. The resulting copolymers exhibit both increased hydrophilicity compared to PPSU, due to the presence of nitrile groups, and superior thermal and mechanical properties compared to similar PAEN copolymers based on DFBN, DCDPS, and bisphenol A.

## Figures and Tables

**Table 1:** Monomer ratios during the synthesis of polymers.

Sample	DHBP/DCDPS/DFBN (mol)
PPSU	0.16/0.1656/0
PPSU/PAEN 75/25	0.16/0.1256/0.04
PPSU/PAEN 50/50	0.16/0.0856/0.08
PPSU/PAEN 25/75	0.16/0.0456/0.12
PAEN	0.16.56/0/0.16

DHBP: 4,4′-dihydroxybiphenyl, DFBN: 2,6-difluorobenzonitrile, DCDPS: 4,4′-dichlorodiphenylsulfone.

**Table 2:** Thermal and rheological properties of PPSU/PAEN copolymers.

Sample	MFI (g min^−1^)	T_2%_	T_5%_	T_10%_	T_g_, °C	T_m_, °C	∆H_m_, J/g
PPSU	28	499	534	564	221	-	-
PPSU/PAEN 75/25	27	497	532	552	218	-	-
PPSU/PAEN 50/50	28	498	532	556	216	-	-
PPSU/PAEN 25/75	44	487	531	554	209	357	10.76
PAEN	6	483	520	549	214	358/371	42.73

MFI: melt flow index, T_2%_, T_5%_, T_10%_: temperature of 2, 5 and 10% weight loss, respectively.

**Table 3:** Solubility of polymers and copolymers in various solvents at 23 °C.

Solvent	PPSU	PPSU/PAEN 75/25	PPSU/PAEN 50/50	PPSU/PAEN 25/75	PAEN
1 h					
N-Methyl-2-pyrrolidone	+ ^1^/+ ^2^	+/−	+/−	−/−	−/−
Dimethylacetamide	+/+	+/−	+/−	−/−	−/−
Dimethylformamide	+/+	+/−	+/−	−/−	−/−
Tetrahydrofuran	+/+	+/−	+/−	−/−	−/−
Methylene chloride	+/+	+/−	+/−	−/−	−/−
Chloroform	+/+	+/−	+/−	−/−	−/−
Styrene	−/−	−/−	−/−	−/−	−/−
Dichloroacetic acid	+/+	+/−	+/−	−/−	−/−
8 h					
N-Methyl-2-pyrrolidone	+/+	+/+	+/−	−/−	−/−
Dimethylacetamide	+/+	+/+	+/+	−/±	−/±
Dimethylformamide	+/+	+/+	+/+	−/±	−/±
Tetrahydrofuran	+/+	+/±	+/−	−/−	−/−
Methylene chloride	+/+	+/+	+/+	−/−	−/−
Chloroform	+/+	+/+	+/+	−/−	−/−
Styrene	−/−	−/−	−/−	−/−	−/−
Dichloroacetic acid	+/+	+/−	+/−	−/−	−/−
24 h					
N-Methyl-2-pyrrolidone	+/+	+/+	+/+	−/+	−/−
Dimethylacetamide	+/+	+/+	+/+	−/+	−/±
Dimethylformamide	+/+	+/+	+/+	−/±	−/±
Tetrahydrofuran	+/+	+/+	+/+	−/−	−/−
Methylene chloride	+/+	+/+	+/+	−/−	−/−
Chloroform	+/+	+/+	+/+	−/−	−/−
Styrene	−/−	−/−	−/−	−/−	−/−
Dichloroacetic acid	+/+	+/−	+/−	−/−	−/−

^1^ powder. ^2^ granules. − did not dissolve, + dissolved, ± swollen

**Table 4:** Mechanical properties of PPSU/PAEN copolymers.

Sample	Impact Strength, kJ/m^2^		E_fl_	σ_bend_	σ_yield_	σ_ten_	ε
	Unnotched	Notched	GPa	MPa	MPa	MPa	%
PPSU	135.0	22.0	2.4	74.5	74.5	58	18.0
PPSU/PAEN 75/25	129.2	18.1	2.4	74.0	74.1	59	13.4
PPSU/PAEN 50/50	103.5	12.0	2.6	76.8	(79.6)	69	13.0
PPSU/PAEN 25/75	75.4	8.6	2.8	82.9	-	83	12.9
PAEN	72.9	9.9	3.0	91.8	-	123	13.1

E_fl_: flexural modulus, σ_yield_: yield strength, σ_bend_: bending strength, σ_ten_: tensile strength, and ε: elongation at break.

## Data Availability

The original contributions presented in this study are included in the article. Further inquiries can be directed to the corresponding author.

## Abbreviations

The following abbreviations are used in this manuscript:

DCDPS	4,4′-dichlorodiphenyl sulfone
DFBN	2,6-difluorobenzonitrile
DHBP	4,4′-dihydroxybiphenyl
DSC	Differential scanning calorimetry
MFI	Melt flow index
PAEN	Polyarylene ether nitrile
PES	Polyethersulfone
PPSU	Polyphenylene sulfone
PSF	Polysulfone
TGA	Thermogravimetric analysis
